# Analysis of changes in heart rate variability after prolonged ultra-high plateau residence in young healthy population: a cross-sectional study

**DOI:** 10.3389/fphys.2025.1529398

**Published:** 2025-03-19

**Authors:** Hongyang Zhang, Hao Liu, Meiting Gong, Xianglin Ye, Peng Wang, Meiling Li, Haixia Yang, Haifeng Pei

**Affiliations:** ^1^ Department of Cardiology, The Affiliated Hospital, Southwest Medical University, Luzhou, China; ^2^ Department of Cardiology, The General Hospital of Western Theater Command, Chengdu, China; ^3^ School of Clinical Medicine, Southwest Jiaotong University, Chengdu, China; ^4^ Department of Pediatrics, The General Hospital of Western Theater Command, Chengdu, China

**Keywords:** heart rate variability, ultra-high plateau, autonomic nervous system, heart rate, sleep

## Abstract

**Objective:**

This study aims to investigate changes in the autonomic nervous system (ANS) by analyzing the characteristics of heart rate variability (HRV).

**Methods:**

A portable 3-lead dynamic electrocardiogram monitoring device was used to collect HRV data from the participants. Based on the inclusion and exclusion criteria, a total of 52 volunteers from the Xinjiang Hetian area (ultra-high plateau group, approximately 5300 m altitude) and 56 volunteers from the Sichuan Chengdu area (plain group, approximately 500 m altitude) were enrolled for the 24-hour long-term HRV data collection. A cross-sectional comparison was made between the groups in terms of various HRV time-domain, frequency-domain, and nonlinear indices. The diurnal and nocturnal variations in HRV and ANS after prolonged residence in the ultra-high plateau were further explored by dividing the day into daytime and nighttime periods and calculating the ΔHRV values. Additionally, the participants’ heart rate and sleep conditions were analyzed.

**Results:**

Compared to the plain group, the ultra-high plateau group showed a significant reduction in overall HRV, with decreased indices of vagal activity (RMSSD, NN50, pNN50, HF, HF norm, and SD1) and increased indices of sympathetic activity (LF norm). The ANS balance indices were increased (LF/HF) and decreased (SD1/SD2), respectively. More importantly, although the diurnal and nocturnal trends of various HRV indices in the ultra-high plateau group were consistent with the plain group, the △HRV value analysis indicated that the ultra-high plateau group had increased △LF (95% CI: 10.20 to 271.60, *P* = 0.031) and △LF/HF (95% CI: −2.23 to −0.49, *P* < 0.001), and decreased △HF (95% CI: −383.10 to −35.50, *P* = 0.012) and △S (95% CI: −12149.47 to −2759.29, *P* = 0.001). Additionally, in the ultra-high plateau group, both the mean and minimum heart rates were elevated compared to the plain group (84.67 ± 1.37 vs. 73.2 ± 0.93 beats/min and 52.9 ± 1.37 vs. 47.57 ± 0.73 beats/min, respectively, *P* < 0.001), while the maximum heart rate was reduced (135.21 ± 1.63 vs. 144.43 ± 3.22 beats/min, *P* = 0.012). Furthermore, the ultra-high plateau group had a significant increase in the number of awakenings (18.27 ± 1.14 vs. 15.34 ± 1.43, *P* = 0.046) and the Apnea-Hypopnea Index (AHI) (20.14 ± 2.47 vs. 11.36 ± 0.76, *P* < 0.001).

**Conclusion:**

Prolonged residence in the ultra-high plateau reduces HRV, cardiac reserve capacity, and sleep quality in healthy young adults, diminishes the diurnal recovery capacity of the vagal nerve, and leads to a shift in ANS balance towards reduced vagal activity and enhanced sympathetic activity.

## 1 Introduction

The extremely high-altitude environment, marked by sparse oxygen and reduced partial pressure of oxygen, often leads to a range of discomforts, such as headaches, reduced appetite, and breathing difficulties among those from lower altitudes living in these regions, potentially triggering various systemic diseases (Hackett and Roach, 2001; Naeije, 2010; Swenson, 2013). It is widely recognized that high-altitude environments impact the function of the autonomic nervous system (ANS) (Billman, 2011), and the ANS plays a significant role in the regulation of cardiovascular functions (Malpas, 2010; Esler, 2000). Monitoring ANS alterations can aid in the early detection of diseases related to high altitudes, leading to the implementation of effective preventative measures. Previous research has indicated that a rapid ascent to a high-altitude area of 3180 m results in a suppression of autonomic nervous activity, with a relative dominance of sympathetic nerve activity (Chen et al., 2008). Lundby et al. have observed that both individuals from plains and native highlanders consistently exhibit increased sympathetic nervous system activity in high-altitude environments (Lundby et al., 2018). However, the patterns of ANS changes in ultra-high plateau environments are not yet fully understood and warrant further research.

The time intervals between successive heartbeats in a healthy heart are subject to continuous fluctuation; these variations are indicative of Heart rate variability (HRV) (Shaffer and Ginsberg, 2017). Importantly, the activity of the ANS is a crucial factor in regulating HRV. In recent years, following the standardization of quantitative indicators for HRV by the “European Society of Cardiology and the North American Society of Pacing and Electrophysiology”, an increasing number of scholars have been utilizing HRV as a means to detect and evaluate the functional activity of the ANS in humans (Author anonymous, 1996; Zaza and Lombardi, 2001). As a non-invasive method, the convenience and reliability of HRV enable doctors to assess the function of the ANS more rapidly and economically, particularly in high-altitude environments where experimental instruments and equipment are lacking. Dhar and colleagues conducted a cross-sectional analysis on the autonomic nervous activity of subjects from plains at altitudes >3500m, finding that altitude significantly affects the HRV and ANS function balance in people from plains (Dhar et al., 2018). However, research is notably scarce on the ultra-high plateau environment, particularly above 5000 m, and the impact of such extreme altitudes on HRV in young healthy populations demands urgent exploration.

This study aims to conduct a cross-sectional investigation through a substantial research cohort on the patterns of HRV in young individuals who have stayed long-term in the ultra-high plateau environment, in order to reveal the characteristics of changes in autonomic nervous function, cardiac and sleep aspects. We hypothesize that prolonged exposure to ultra-high plateau environments may reduce heart rate variability, leading to decreased vagal tone, increased sympathetic tone, and impaired sleep quality in this population.

## 2 Methods

### 2.1 Ethical statement

This study, conducted from March to July 2023, recruited 66 healthy young volunteers from the Hetian area of Xinjiang (approximately 5300 m above sea level), the region spans longitude 76°08′-76°30′E and latitude 35°28′-38°34′N and 70 healthy young volunteers from the Chengdu area in Sichuan (approximately 500 m above sea level), the region spans longitude 102°54′-104°53′E and latitude 30°05′-31°26′N. All participants were informed about the purpose and procedure of the study and signed informed consent forms. The research protocol adhered to the Declaration of Helsinki for human experimentation and was approved by the Ethics Committee of the Western Theater Command General Hospital (Registration Number: 2022EC2-Ky051).

### 2.2 Dynamic electrocardiogram data collection

In this study, HRV measures were obtained using a Patch-type electrocardiogram (ECG) from a wearable long-term Holter monitoring patch (401 three electrodes patch-type Holter, Chengdu, China). ECGs signal-lead were measured in the middle of the chest location, The ECG signal was digitally stored and trans mitted to a cloud storage center. Each participant began wearing the device at 8:00 AM and removed it at 8:00 AM the following day, with the data uploaded to cloud storage. Data analysis was performed using the Modular ECG analysis system and manual assessment by two trained technicians to interpret the HRVs and ECGs. The data for each participant were divided into a daytime period (8:00 AM to 8:00 PM) and a nighttime period (8:00 PM to 8:00 AM the next day) for analysis of diurnal variations. △HRVs (the difference between the nighttime period HRV value minus that of the daytime period) were used to describe the diurnal regulation capacity of each HRV index. The calculation and analysis method for △HRV has been utilized in numerous studies (Nicolini et al., 2022; Rodríguez-Carbó et al., 2022), effectively demonstrates the magnitude of HRV variations. The average heart rate, maximum heart rate, and minimum heart rate for each participant were directly obtained from the final cleaned data. Although the dynamic ECG recording time standard proposed by the “European Society of Cardiology and the North American Society for Pacing and Electrophysiology Working Group” in 1996 suggests that ≥18 h is sufficient for data validity (Author anonymous, 1996), to control potential impacts of measurement duration, we limited this period to 20–24 h. Participants carried on with their normal activities while wearing the device to capture data that closely resembles their natural living conditions. Time-domain indicators are calculated directly from the original RR interval time series, including SDNN (the standard deviation of the normal-to-normal intervals), RMSSD (the root mean square of successive differences between normal heartbeats), NN50 (the number of adjacent NN intervals that differ from each other by more than 50 ms), pNN50 (percentage of successive NN intervals that differ by more than 50 ms), SDANN (the standard deviation of the average NN intervals for each of the 5 min segments during a 24 h recording), and SDNNI (the mean of the standard deviations of all the NN intervals for each 5 min segment of a 24-h HRV recording). Frequency-domain indicators, derived using the Fast Fourier Transform (FFT) method to calculate the power spectral density of the RR sequence, include total power of NN intervals (TP, 0–0.4 Hz), very-low-frequency band (VLF, 0.0033–0.04 Hz), low-frequency band (LF, 0.04–0.15 Hz), high-frequency band (HF, 0.15–0.4 Hz), ratio of LF-to-HF power (LF/HF), and the normalized values LF norm = LF×100/(TP−VLF) and HF norm = HF×100/(TP−VLF). Geometric measures of HRV such as the HRV triangular index (HTI) and TINN (triangular Interpolation of the NN Interval Histogram) are calculated. Finally, analysis of the Poincaré plot by fitting an ellipse (similar to a flattened circle) yields three nonlinear measurements: S (area of the ellipse which represents total HRV), SD1 (Poincaré plot standard deviation perpendicular the line of identity), SD2 (Poincaré plot standard deviation along the line of identity), and the ratio SD1/SD2. In addition, indices used for analyzing chaotic systems - DFAα1 (detrended fluctuation analysis, describes short-term fluctuations), DFAα2 (detrended fluctuation analysis, describes long-term fluctuations), ApEn (approximate entropy), and SampEn (sample entropy) - are also incorporated in this study.

### 2.3 Inclusion and exclusion criteria for participants

**FIGURE 1:**
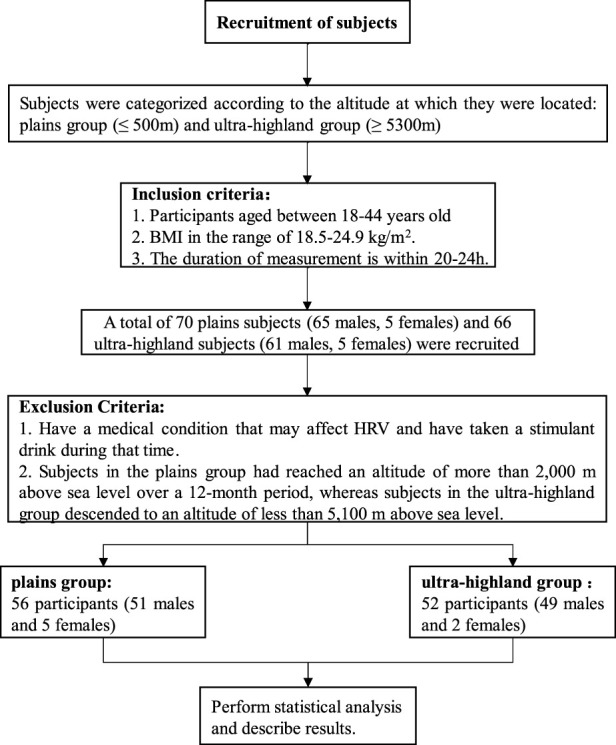
Participant inclusion and exclusion process.

As shown in Figure 1, the inclusion criteria for participants in this study are: (1) Age between 18 and 44 years; (2) Body Mass Index (BMI) within the normal range defined by WHO (18.5–24.9 kg/m^2^); (3) Duration of dynamic electrocardiogram data between 20 and 24 h. The exclusion criteria include: (1) Suffering from hypertension, diabetes, asthma, chronic pain, allergies, mental illnesses (stress, anxiety, depression), heart diseases, neoplasms, endocrinopathies, other metabolic disorders that are considered to cause vasovagal syndrome and taking medication that affects the ANS or consuming stimulants like coffee during the measurement period; (2) For participants in the plain group, having reached elevations above 2000m in the past 12 months, and for participants in the ultra-high plateau group, having descended below 5100 m. Ultimately, the study included 24-hour dynamic ECG data from 56 participants in the plain area (500 m, 12 months) and 52 participants in the ultra-high plateau area (5300 m, 12 months).

### 2.4 Statistical analysis

Initially, SPSS 27.0 statistical software is used to test the normality of data from both groups. The Shapiro-Wilk test was used to assess normality, if the data follow a normal distribution, the independent samples Student's t-test is chosen; otherwise, the Mann-Whitney U test is used for difference testing between the two groups. The paired-samples Student's t-test or Wilcoxon test was applied to examine the differences within groups at various time points. All HRV data in the text are presented as mean ± standard error of the mean (SEM). Other quantitative data, if normally distributed, are expressed as mean ± standard deviation (SD). A *P*-value of <0.05 (two-tailed) will be considered statistically significant.

## 3 Results

### 3.1 Physiological characterization of subjects in the ultra-high plateau and plain groups

The study ultimately included 56 subjects from the plain group and 52 from the ultra-high plateau group. The age span of individuals in the plains group was 19–38 years (mean age 24.2 ± 3.76 years), with a composition of 91% male and 95% belonging to the Han ethnic group. Their Body Mass Index (BMI) varied from 18.6 to 24.9 kg/m^2^ (mean BMI 21.95 ± 1.83 kg/m^2^). In the ultra-high plateau group, ages ranged from 20 to 39 years (mean age 26.04 ± 5.08 years), with 94% male and 99% of Han ethnicity. The BMI distribution in this group was between 18.5 and 24.9 kg/m^2^ (mean BMI 21.22 ± 1.84 kg/m^2^). As indicated in Table 1, analysis revealed no significant discrepancies in age, gender, and ethnic composition between the ultra-high plateau and plain groups (*P* > 0.05). Although the ultra-high plateau group exhibited a marginally lower BMI compared to the plain group (*P* = 0.041), the values for both groups were within normal ranges. This data suggests that the baseline physiological characteristics of the subjects in both groups are fundamentally consistent, adhering to the expected parameter range for this study.

**TABLE 1:** Basic Physiological Profiles of Young Healthy Subjects in Ultra-highland and Plain groups.*

Characteristic	Plain group (n = 56)	Ultra-high plateau group (n = 52)	P Value
Age — yr			
Mean	24.2 ± 3.76	26.04 ± 5.08	0.106
Range	19–38	20–39	
Male sex — no. (%)	51 (91%)	49 (94%)	0.531
Ethnic Han — no. (%)	53 (95%)	51 (99%)	0.345
BMI† — kg/m^2^	21.95 ± 1.83	21.22 ± 1.84	0.041

* Plus–minus values are means ± SD., Percentages may not total 100 because of rounding. There were no significant differences in baseline data between the two groups, except for BMI (*P* = 0.041).

† The body mass index (BMI) is the weight in kilograms divided by the square of the height in meters.

### 3.2 Prolonged residence at an ultra-high plateau reduces heart rate variability in the young healthy population

As shown in Table 2, there were significant differences in all HRV indices between the two groups (*P* < 0.05). Relative to the plain group, the ultra-high plateau group exhibited a pronounced reduction in overall HRV indices (including SDNN, TINN, and TP) and markers of parasympathetic nervous activity (such as RMSSD, NN50, pNN50, HF, HF norm, and SD1) (*P* < 0.001). In contrast, the indicator of sympathetic nervous activity (LF norm) was significantly elevated (*P* < 0.001). The metrics representing ANS balance (LF/HF and SD1/SD2) showed a significant rise (*P* < 0.001) and a notable fall (*P* = 0.017), respectively. Furthermore, when compared with the plains group, the remaining HRV indices in the ultra-high plateau group (including HTI, SDANN, SDNNI, VLF, LF, S, SD2, DFAα2, ApEn, and SampEn) also demonstrated significant reductions (*P* < 0.05). These findings suggest that a prolonged stay in an ultra-high plateau environment substantially diminishes the overall HRV in the young healthy population, with changes in autonomic nervous function characterized by a significant decrease in parasympathetic nervous tension accompanied by a significant increase in sympathetic nervous tension.

**TABLE 2:** Analysis of Heart rate variability in Young Healthy Subjects between Ultra-High Plateau and Plain Groups.*

Parameters	Plain group (n = 56)	Ultra-high plateau group (n = 52)	Statistics	95% CI of the difference	P value
Time-domain					
SDNN (ms)	190.11 ± 5.12	145.88 ± 5.54	5.506‡	45.35 (32.10–58.5)	<0.001
RMSSD (ms)	46.37 ± 2.24	31.74 ± 1.74	4.593‡	13.65 (8.00–20.00)	<0.001
NN50	19754.82 ± 1286.48	11052.35 ± 1004.10	4.636‡	8872.00 (5374.00–12498.00)	<0.001
pNN50 (%)	20.31 ± 1.50	9.88 ± 0.96	5.057‡	9.98 (6.25–13.92)	<0.001
HTI	47.07 ± 1.35	32.02 ± 1.17	8.365†	1.60 (1.16–2.02)	<0.001
TINN (ms)	729.49 ± 24.90	476.12 ± 20.43	7.865†	1.49 (1.06–1.91)	<0.001
SDANN (ms)	176.67 ± 5.28	139.30 ± 5.84	4.811‡	38.35 (24.50–52.30)	<0.001
SDNNI (ms)	74.08 ± 2.00	56.82 ± 2.02	6.06†	1.15 (0.75–1.56)	<0.001
Frequency-domain					
VLF (ms^2^)	18022.68 ± 858.11	11386.00 ± 1102.33	5.359‡	7227.85 (4904.30–9700.9)	<0.001
LF (ms^2^)	1271.68 ± 80.50	1040.67 ± 78.22	2.029‡	232.00 (12.60–444.30)	0.042
HF (ms^2^)	895.47 ± 82.09	390.59 ± 54.86	5.119‡	413.20 (248.80–599.60)	<0.001
TP (ms^2^)	20255.66 ± 927.96	12876.40 ± 1163.67	5.478‡	8068.30 (5501.20–10656.50)	<0.001
LF/HF	1.86 ± 0.13	3.75 ± 0.30	5.577‡	−1.66 (−2.20 to −1.15)	<0.001
LF norm	59.42 ± 1.47	71.34 ± 1.49	5.159‡	−13.20 (−17.40 to −8.50)	<0.001
HF norm	37.30 ± 1.46	23.94 ± 1.48	5.706‡	14.65 (10.40–18.70)	<0.001
Non-linear					
S (ms^2^)	28826.54 ± 1975.27	15610.83 ± 1484.64	5.325‡	12009.17 (7514.20–16703.44)	<0.001
SD1 (ms)	32.78 ± 1.58	22.44 ± 1.23	4.605‡	9.65 (5.70–14.10)	<0.001
SD2 (ms)	266.73 ± 7.16	204.99 ± 7.78	5.515‡	63.05 (45.00–81.70)	<0.001
SD1/SD2	0.121 ± 0.004	0.109 ± 0.004	2.391‡	0.014 (0–0.028)	0.017
DFAα1	1.122 ± 0.022	1.268 ± 0.022	4.375‡	−0.163 (−0.226 to −0.093)	<0.001
DFAα2	1.119 ± 0.008	1.068 ± 0.011	3.871‡	0.048 (0.026–0.073)	<0.001
ApEn	0.710 ± 0.022	0.614 ± 0.024	3.08‡	0.105 (0.040–0.171)	0.002
SampEn	0.481 ± 0.021	0.363 ± 0.020	4.166‡	0.127 (0.072–0.183)	<0.001

* Plus–minus values are means ± SEM., All heart rate variability data were retained to two decimal places except for SD1/SD2, DFAα1, DFAα2, ApEn and SampEn data, which were rounded to three decimal places. SDNN, denotes the standard deviation of the normal-to-normal intervals, RMSSD, the root mean square of successive differences between normal heartbeats, NN50 the number of adjacent NN, intervals that differ from each other by more than 50 ms, pNN50 percentage of successive NN, intervals that differ by more than 50 ms, HTI, integral of the density of the NN, interval histogram divided by its height, TINN, triangular interpolation of the NN, interval histogram, SDANN, the standard deviation of the average NN, intervals for each of the 5 min segments during a 24 h recording, SDANNI, the mean of the standard deviations of all the NN, intervals for each 5 min segment of a 24-h HRV, recording, VLF, very-low-frequency band (0.0033–0.04 Hz), LF, low-frequency band (0.04–0.15 Hz), HF, high-frequency band (0.15–0.4 Hz), TP, total power of NN, intervals (0–0.4 Hz), LF/HF, ratio of LF-to-HF, power, LF, norm LF×100/(TP − VLF), HF, norm HF × 100/(TP − VLF), S area of the ellipse which represents total HRV, SD1 Poincaré plot standard deviation perpendicular the line of identity, SD2 Poincaré plot standard deviation along the line of identity, SD1/SD2 ratio of SD1-to-SD2, DFAα1 detrended fluctuation analysis (describes short-term fluctuations), DFAα2 detrended fluctuation analysis (describes long-term fluctuations), ApEn approximate entropy and SampEn Sample entropy.

† Statistics was calculated with the Student-t test.

‡ Statistics was calculated with the Mann-Whitney U test.

### 3.3 The autonomic nervous system’s diurnal regulation ability is diminished in young healthy population residing long-term at ultra-high plateaus

Table 3 shows the analysis results of the diurnal changes in all HRV indices. The ultra-high plateau day group, in comparison to the plain day group, demonstrated a significant decrease in overall HRV indices (SDNN, TINN, TP) and markers of parasympathetic nervous activity (RMSSD, NN50, pNN50, HF, HF norm, SD1) (*P* < 0.001). Conversely, the sympathetic nervous activity marker (LF norm) showed a significant increase (*P* < 0.001). Furthermore, the indicators of ANS balance (LF/HF and SD1/SD2) exhibited a significant rise (*P* < 0.001) and fall (*P* = 0.046), respectively. In addition, compared to the plain day group, the remaining HRV study variables in the ultra-high plateau day group (such as HTI, SDANN, SDNNI, VLF, LF, S, SD2, DFAα2, ApEn, and SampEn) were significantly reduced (*P* < 0.05). Moreover, the changes in the ultra-high plateau night group were consistent with those observed during the day, except for the indices LF, SD1/SD2, ApEn, and SampEn, which did not show a difference (*P* > 0.05). This suggests that in young healthy individuals living long-term at ultra-high plateaus, HRV is consistently reduced across diurnal and nocturnal periods, with ANS adaptations marked by decreased parasympathetic and increased sympathetic nervous activity.

**TABLE 3:** Analysis of diurnal and nocturnal characteristics of heart rate variability in young healthy subjects in ultra-high plateau and plain Areas.*

Parameters	Plain day group (n = 56)	Plain night group (n = 56)	Ultra-high plateau day group (n = 52)	Ultra-high plateau night group (n = 52)	P value			
					Plain day group vs. Plain night group	Ultra-high plateau day group vs. Ultra-high plateau night group	Ultra-high plateau day group vs. Plain day group	Ultra-high plateau night group vs. Plain night group
Time-domain								
SDNN(ms)	139.03 ± 4.23	199.96 ± 6.84	97.39 ± 4.08	144.72 ± 6.11	<0.001	<0.001	<0.001	<0.001
RMSSD (ms)	37.96 ± 1.88	55.63 ± 2.90	23.95 ± 1.29	38.87 ± 2.49	<0.001	<0.001	<0.001	<0.001
NN50	7408.27 ± 603.25	11890.82 ± 733.53	2779.27 ± 328.27	7375.96 ± 685.86	<0.001	<0.001	<0.001	<0.001
pNN50(%)	15.23 ± 1.39	27.69 ± 1.88	5.65 ± 0.72	15.37 ± 1.56	<0.001	<0.001	<0.001	<0.001
HTI	36.12 ± 1.09	45.06 ± 1.79	23.68 ± 0.84	32.52 ± 1.38	<0.001	<0.001	<0.001	<0.001
TINN(ms)	564.18 ± 19.34	732.99 ± 33.83	355.93 ± 13.67	535.47 ± 28.66	<0.001	<0.001	<0.001	<0.001
SDANN(ms)	118.82 ± 4.49	170.23 ± 6.26	86.49 ± 4.62	124.63 ± 5.58	<0.001	<0.001	<0.001	<0.001
SDNNI (ms)	68.86 ± 1.99	79.93 ± 2.33	49.29 ± 1.78	63.91 ± 2.62	<0.001	<0.001	<0.001	<0.001
Frequency-domain								
VLF (ms^2^)	12399.66 ± 691.71	17237.4 ± 904.8	6497.26 ± 611.49	10242.99 ± 930.79	<0.001	<0.001	<0.001	<0.001
LF (ms^2^)	1190.95 ± 79.82	1376.85 ± 96.32	889.43 ± 75.79	1273.99 ± 125.17	0.014	<0.001	0.002	0.197
HF (ms^2^)	566.85 ± 53.88	1262.86 ± 127.16	209.24 ± 29.06	573.49 ± 92.2	<0.001	<0.001	<0.001	<0.001
TP (ms^2^)	14232.13 ± 773.7	19934.59 ± 1003.82	7650.95 ± 665.99	11932.76 ± 1058.72	<0.001	<0.001	<0.001	<0.001
LF/HF	2.79 ± 0.21	1.49 ± 0.12	5.75 ± 0.35	3.25 ± 0.35	<0.001	<0.001	<0.001	<0.001
LF norm	66.81 ± 1.42	54.29 ± 1.62	78.06 ± 1.13	67.76 ± 1.74	<0.001	<0.001	<0.001	<0.001
HF norm	28.87 ± 1.34	43.17 ± 1.63	16.51 ± 1.05	28.00 ± 1.73	<0.001	<0.001	<0.001	<0.001
Non-linear								
S (ms^2^)	17128.11 ± 1232.85	36731.18 ± 2803.19	7900.32 ± 753.75	19294.77 ± 2205.71	<0.001	<0.001	<0.001	<0.001
SD1 (ms)	26.84 ± 1.32	39.39 ± 2.04	16.95 ± 0.91	27.47 ± 1.76	<0.001	<0.001	<0.001	<0.001
SD2 (ms)	193.42 ± 6.02	279.79 ± 9.58	137.24 ± 5.75	202.65 ± 8.53	<0.001	<0.001	<0.001	<0.001
SD1/SD2	0.139 ± 0.005	0.14 ± 0.006	0.124 ± 0.005	0.13 ± 0.006	0.718	0.059	0.046	0.440
DFAα1	1.225 ± 0.021	1.05 ± 0.02	1.372 ± 0.019	1.21 ± 0.02	<0.001	<0.001	<0.001	<0.001
DFAα2	1.088 ± 0.009	1.14 ± 0.008	1.022 ± 0.013	1.09 ± 0.01	<0.001	<0.001	<0.001	<0.001
ApEn	0.830 ± 0.029	0.81 ± 0.03	0.740 ± 0.029	0.77 ± 0.03	0.166	0.321	0.046	0.389
SampEn	0.611 ± 0.028	0.61 ± 0.03	0.530 ± 0.026	0.55 ± 0.03	0.530	0.636	0.038	0.165

* Plus–minus values are means ± SEM., All heart rate variability data were retained to two decimal places except for SD1/SD2, DFAα1, DFAα2, ApEn and SampEn data, which were rounded to three decimal places. SDNN, denotes the standard deviation of the normal-to-normal intervals, RMSSD, the root mean square of successive differences between normal heartbeats, NN50 the number of adjacent NN, intervals that differ from each other by more than 50 ms, pNN50 percentage of successive NN, intervals that differ by more than 50 ms, HTI, integral of the density of the NN, interval histogram divided by its height, TINN, triangular interpolation of the NN, interval histogram, SDANN, the standard deviation of the average NN, intervals for each of the 5 min segments during a 24 h recording, SDANNI, the mean of the standard deviations of all the NN, intervals for each 5 min segment of a 24-h HRV, recording, VLF, very-low-frequency band (0.0033–0.04 Hz), LF, low-frequency band (0.04–0.15 Hz), HF, high-frequency band (0.15–0.4 Hz), TP, total power of NN, intervals (0–0.4 Hz), LF/HF, ratio of LF-to-HF, power, LF, norm LF×100/(TP − VLF), HF, norm HF × 100/(TP − VLF), S area of the ellipse which represents total HRV, SD1 Poincaré plot standard deviation perpendicular the line of identity, SD2 Poincaré plot standard deviation along the line of identity, SD1/SD2 ratio of SD1-to-SD2, DFAα1 detrended fluctuation analysis (describes short-term fluctuations), DFAα2 detrended fluctuation analysis (describes long-term fluctuations), ApEn approximate entropy and SampEn Sample entropy.

Compared to the plain day group, the plain night group showed a significant increase in overall HRV indices (SDNN, TINN, and TP) and parasympathetic nervous tension indicators (RMSSD, NN50, pNN50, HF, HF norm, SD1) (*P* < 0.001), while the sympathetic nervous tension indicator (LF norm) and the autonomic nervous function balance indicator (LF/HF) significantly decreased (*P* < 0.001). In addition, compared to the plain day group, the remaining HRV research variables in the plain night group (such as HTI, SDANN, SDNNI, VLF, LF, S, SD2, DFAα2) significantly increased (*P* < 0.05). Importantly, the diurnal and nocturnal changes in HRV indices in the ultra-high plateau group were consistent with those in the plain group. To observe whether the ultra-high plateau environment affects the amplitude of diurnal and nocturnal changes in HRV indices, we analyzed the △HRV values, i.e., the difference between the night group and the corresponding day group HRV indices for both plain and ultra-high plateau groups. As shown in Table 4, compared to the plain group, the △LF (P = 0.031) and △LF/HF (*P* < 0.001) in the ultra-high plateau group significantly increased, while △HF (*P* = 0.012) and △S (*P* = 0.001) significantly decreased. In conclusion, long-term residence in the ultra-high plateau environment significantly reduces the diurnal and nocturnal autonomic nervous regulation ability in young healthy individuals, characterized by a reduced recovery ability of parasympathetic nervous tension and a continuous increase in sympathetic nervous activity tension.

**TABLE 4:** Analysis of Diurnal and Nocturnal Amplitude Variations of Heart rate variability in Young Healthy Subjects of Ultra-High Plateau and Plain Groups.*

Parameters	Plain group (n = 56)	Ultra-high plateau group (n = 52)	Statistics	95% CI of the difference	P value
Time-domain					
∆SDNN(ms)	60.93 ± 6.10	47.33 ± 5.30	−1.673†	−13.60 (−29.73 to 2.52)	0.097
∆RMSSD (ms)	17.68 ± 1.78	14.91 ± 1.72	1.104‡	−2.45 (−7.10 to 2.20)	0.270
∆NN50	4482.55 ± 440.08	4596.69 ± 498.97	0.172†	114.14 (−1200.57–1428.85)	0.864
∆pNN50(%)	12.46 ± 1.14	9.72 ± 1.18	1.881‡	−3.02 (−6.29 to 0.15)	0.060
∆HTI	8.94 ± 1.71	8.84 ± 1.16	−0.044†	−0.093 (−4.26 to 4.07)	0.965
∆TINN(ms)	168.82 ± 34.77	179.54 ± 26.01	0.244†	10.73 (−76.39–97.84)	0.808
∆SDANN(ms)	51.41 ± 6.29	38.13 ± 5.46	−1.585†	−13.29 (−29.91 to 3.34)	0.116
∆SDNNI (ms)	11.06 ± 1.42	14.62 ± 1.73	−1.842‡	4.00 (−0.20–7.80)	0.066
Frequency-domain					
∆VLF (ms^2^)	4837.74 ± 917.02	3745.73 ± 858.00	−0.866†	−1092.02 (-3591.44 to 1407.41)	0.388
∆LF (ms^2^)	185.91 ± 63.50	384.57 ± 103.48	−2.161‡	142.30 (10.20–271.60)	0.031
∆HF (ms^2^)	696.01 ± 95.43	364.25 ± 70.83	2.515‡	−181.20 (−383.10 to −35.50)	0.012
∆TP (ms^2^)	5702.46 ± 947.73	4281.82 ± 927.27	−1.069†	−1420.65 (−4055.16 to 1213.87)	0.287
∆LF/HF	−1.31 ± 0.15	−2.50 ± 0.34	3.317‡	−1.285 (−2.23 to −0.49)	<0.001
∆LF norm	−12.52 ± 1.20	−10.30 ± 1.45	1.188†	2.22 (−1.48–5.92)	0.237
∆HF norm	14.31 ± 1.24	11.49 ± 1.45	−1.475†	−2.81 (−6.59 to 0.97)	0.143
Non-linear					
∆S (ms^2^)	19603.07 ± 1966.46	11394.46 ± 1717.34	3.240‡	−7162.535 (−12149.47 to −2759.29)	0.001
∆SD1 (ms)	12.55 ± 1.25	10.53 ± 1.22	1.159‡	−1.80 (−5.00 to 1.40)	0.246
∆SD2 (ms)	86.38 ± 8.55	65.41 ± 7.47	−1.836†	−20.97 (−43.48 to 1.53)	0.069
∆SD1/SD2	0.001 ± 0.005	0.010 ± 0.005	−1.755‡	0.01 (0–0.02)	0.079
∆DFAα1	−0.177 ± 0.018	−0.159 ± 0.021	0.668†	0.018 (−0.036–0.073)	0.505
∆DFAα2	0.055 ± 0.007	0.072 ± 0.011	1.343†	0.017 (−0.008–0.042)	0.182
∆ApEn	−0.024 ± 0.032	0.032 ± 0.026	1.336†	0.056 (−0.026–0.138)	0.185
∆SampEn	0.002 ± 0.033	0.023 ± 0.026	−0.965‡	0.038 (−0.036–0.116)	0.334

* Plus–minus values are means ± SEM., All heart rate variability data were retained to two decimal places except for SD1/SD2, DFAα1, DFAα2, ApEn and SampEn data, which were rounded to three decimal places. △ denotes The difference between the nighttime period HRV, value minus that of the daytime period, SDNN, the standard deviation of the normal-to-normal intervals, RMSSD, the root mean square of successive differences between normal heartbeats, NN50 the number of adjacent NN, intervals that differ from each other by more than 50 ms, pNN50 percentage of successive NN, intervals that differ by more than 50 ms, HTI, integral of the density of the NN, interval histogram divided by its height, TINN, triangular interpolation of the NN, interval histogram, SDANN, the standard deviation of the average NN, intervals for each of the 5 min segments during a 24 h recording, SDANNI, the mean of the standard deviations of all the NN, intervals for each 5 min segment of a 24-h HRV, recording, VLF, very-low-frequency band (0.0033–0.04 Hz), LF, low-frequency band (0.04–0.15 Hz), HF, high-frequency band (0.15–0.4 Hz), TP, total power of NN, intervals (0–0.4 Hz), LF/HF, ratio of LF-to-HF, power, LF, norm LF×100/(TP − VLF), HF, norm HF × 100/(TP − VLF), S area of the ellipse which represents total HRV, SD1 Poincaré plot standard deviation perpendicular the line of identity, SD2 Poincaré plot standard deviation along the line of identity, SD1/SD2 ratio of SD1-to-SD2, DFAα1 detrended fluctuation analysis (describes short-term fluctuations), DFAα2 detrended fluctuation analysis (describes long-term fluctuations), ApEn approximate entropy and SampEn Sample entropy.

† Statistics was calculated with the Student-t test.

‡ Statistics was calculated with the Mann-Whitney U test.

### 3.4 Prolonged residence at an ultra-high plateau reduces cardiac reserve capacity in the young healthy population

Table 5 presents the impact of long-term exposure to an ultra-high plateau environment on heart rate parameters in a young, healthy population. Compared to the plain group, in the ultra-high plateau group, both the mean and minimum heart rates significantly increased (*P* < 0.001), whereas the maximal heart rate significantly decreased (*P* = 0.012). This suggests that prolonged residence in the ultra-high plateau environment may reduce the cardiac reserve capacity in young healthy individuals.

**TABLE 5:** Analysis of the impact of ultra-high plateau on heart rate in young healthy subjects.*

Parameters	Plain group (n = 56)	Ultra-high plateau group (n = 52)	Statistics	P value
Mean HR (beats/min)	73.2 ± 0.93	84.67 ± 1.37	6.241‡	<0.001
Maxmal HR (beats/min)	144.43 ± 3.22	135.21 ± 1.63	2.555†	0.012
Minimum HR (beats/min)	47.57 ± 0.73	52.9 ± 1.37	3.963‡	<0.001

* Plus–minus values are means ± SD.

† Statistics was calculated with the Student-t test.

‡ Statistics was calculated with the Mann-Whitney U test.

### 3.5 Prolonged residence at an ultra-high plateau reduces the sleep quality in the young healthy population

As shown in Table 6, compared to the plain group, the ultra-high plateau group exhibited a significant increase in the proportion of unstable sleep time, the number of awakenings, and the Apnea-Hypopnea Index (AHI) (*P* < 0.05). In contrast, metrics such as stable sleep duration, overall sleep efficiency, and sleep quality scores were significantly reduced (*P* < 0.05) in the ultra-high plateau group. However, there was no significant statistical difference in the proportion of rapid eye movement (REM) sleep time between the two groups (*P* > 0.05). These results indicate that the ultra-high plateau environment significantly reduces sleep quality in young, healthy populations.

**TABLE 6:** Analysis of sleep conditions among young healthy subjects in ultra-high plateau and plain areas.*

Parameters	Plain group (n = 56)	Ultra-high plateau group (n = 52)	Statistics	P value
Proportion of REM, %	10.76 ± 1.21	11.5 ± 1.19	0.667‡	0.505
Proportion of Unstable Sleep, %	9.01 ± 0.96	15.81 ± 1.58	3.314‡	<0.001
Proportion of Stable Sleep, %	41.55 ± 2.62	26.07 ± 1.9	4.141‡	<0.001
Sleep Efficiency, %	61.31 ± 2.8	52.84 ± 2.39	2.283†	0.024
The Number of Awakenings	15.34 ± 1.43	18.27 ± 1.14	1.997‡	0.046
AHI	11.36 ± 0.76	20.14 ± 2.47	3.462‡	<0.001
Sleep Quality Scores	73.52 ± 1.79	66.58 ± 1.61	2.922‡	0.003

* Plus–minus values are means ± SD.

† Statistics was calculated with the Student-t test.

‡ Statistics was calculated with the Mann-Whitney U test.

## 4 Disscussion

Studies indicate that ascending to high-altitude areas can lead to significant changes in HRVdue to disturbances in the ANS functions (Calbet, 2003; Hainsworth and Drinkhill, 2007). However, the specific changes and characteristics that occur in the youth population from plains who have long resided in ultra-high plateau environments (altitude >5000 m) remain unclear. In this study, we conducted a cross-sectional comparison of HRV and ANS functions in the young population on a diurnal and nocturnal level, and delved deeper into the variations in △HRV. The findings reveal that the ultra-high plateau environment significantly reduces the overall HRV in young healthy individuals. This is characterized by a notable decrease in vagal nerve tension accompanied by a significant increase in sympathetic nerve tension, and these changes do not vary with the day-night cycle. Importantly, the diurnal and nocturnal trends in HRV and ANS functions show consistency between the plain and ultra-high plateau environments. Further analysis of △HRV suggests that prolonged residence in ultra-high plateau environments significantly impairs the diurnal and nocturnal regulatory ability of the ANS in young healthy populations, indicated by a reduced recovery ability of vagal nerve activity and a continuous increase in sympathetic nerve tension. Additionally, the study also demonstrates that prolonged residence in ultra-high plateau environments significantly reduces cardiac reserve capacity and sleep quality in young healthy populations.

The ANS plays a crucial role in regulating the oscillatory behavior of the cardiovascular system. Analyzing time-domain, frequency-domain, and nonlinear characteristics of HRV provides a non-invasive assessment of sympathetic and vagal nerve activities. Extended monitoring duration helps observe the diurnal balance changes of ANS. In HRV analysis, SDNN, TINN, and TP indicate the overall level of HRV. RMSSD, NN50, pNN50, HF, HF norm, and SD1 effectively represent vagal nerve excitability, while LF/HF and SD1/SD2 indicate the balance of ANS activity (Armstrong et al., 2022). LF was once considered a crucial index in HRV to reflect the functional state of the sympathetic nervous system. However, with advancing research, LF is no longer deemed a valid marker of autonomic activity alone, as it is also influenced by baroreceptor activity (Goldstein et al., 2011). Additionally, nonlinear indicators such as ApEn and SampEn are used to measure the regularity and complexity of HRV time series, with lower values indicating greater uniformity in adjacent beat intervals and less fluctuation between heartbeats. The HRV analysis in this study was conducted over a 24-hour period, thus providing a more accurate assessment of long-term ANS function.

In this study, significant reductions were observed in SDNN, TINN, and TP in the ultra-high plateau group compared to the plain group, indicating a substantial decrease in overall HRV in the ultra-high plateau environment. The parasympathetic markers (RMSSD, HF, and SD1) showed a decrease, whereas the sympathetic marker (LF norm) increased. Trends in LF/HF and SD1/SD2 ratios, as indicated in Table 2, reveal a shift towards increased sympathetic nervous activity within the ANS due to prolonged ultra-high plateau exposure. Previous research indicates that rapid ascent to high altitudes causes a decrease in LF and HF power and an increase in the LF/HF ratio (Saito et al., 2005; Cornolo et al., 1985; Kanai et al., 2001). Huang et al. (2010) found that the R-R intervals, LF, and HF power significantly decreased in plains-dwelling subjects within 5 days of trekking up to an altitude of 3440m, compared to sea level. Other research noted declines in SDNN, TP, and HF norm, along with an increase in LF norm and LF/HF ratio, in subjects ascending rapidly to 3180 m (Chen et al., 2008). Similarly, Dhar et al. (2018) reported that prolonged stay at altitudes >3500 m significantly reduced overall HRV indices (SDNN, TINN, and TP) and parasympathetic nervous tension indicators (RMSSD, NN50, pNN50, HF, HF norm, SD1) in lowlanders, while sympathetic nervous tension indicators (LF norm) and the LF/HF ratio significantly increased. It is evident that both rapid ascent and prolonged stay in high-altitude areas lead to significant changes in HRV indices. In this study, the trend of HRV changes in the ultra-high plateau group (see Table 2) is consistent with the results of the aforementioned studies, indicating that even after a 12-month stay, the hypoxic environment of the ultra-high plateau region continues to impact the ANS function of individuals from the plains. It is commonly believed that acute hypoxia leads to enhanced sympathetic nerve activity, primarily due to the activation of carotid bodies and chemoreceptors in the brainstem (Marshall, 1994; Solomon, 2000). Calbet et al. demonstrated significant activation of the sympathetic nervous system under chronic hypoxia by examining the systemic epinephrine and norepinephrine overflow in lowlanders exposed to an altitude of 5260 m for 9 weeks (Calbet, 2003). Recently, Siebenmann et al. conducted a pharmacological blockade experiment at 3454 m and discovered that a reduction in cardiac vagal nerve activity is the primary mechanism for the heart rate increase related to 2 weeks of hypoxia (Siebenmann et al., 2017). The current study’s findings point towards ongoing sympathetic nerve tension activation and a simultaneous decrease in vagal nerve tension, as reflected by HRV indices. The specific factors contributing to the reduced function of the vagal nerve remain to be elucidated. Persistent hypoxia is a significant factor in the development of pulmonary arterial hypertension due to long-term exposure to high altitudes (Penaloza and Arias-Stella, 2007). Reductions in SDNN, LF, LF/HF, and VLF, as observed by Tsai et al. in patients with pulmonary arterial hypertension (Tsai et al., 2019), were also evident in the ultra-high plateau group. These reductions may be early indicators of cardio-pulmonary diseases associated with continuous exposure to an extreme high-altitude environment.

Previous study highlights that HRV in healthy individuals, influenced by diurnal rhythmicity, reflects circadian shifts in ANS function, typically showing greater sympathetic nerve activity during the day and heightened vagal nerve tension at night (Sammito et al., 2016). Nevertheless, the changes in HRV diurnal rhythms within ultra-high plateau environments are still not well-defined. To address this question, the current study segmented data from both ultra-high plateau and plain groups into daytime and nighttime periods for comparative analysis (refer to Table 3). The findings indicate that, when comparing the ultra-high plateau group with the plain group during corresponding day and night periods, the trends in HRV indices were generally in line with the overall 24-h variation observed. Notably, the analysis of day and night periods within the same group reveals that both groups exhibited increased HRV at night, suggesting reduced sympathetic and enhanced vagal nerve activity. The aforementioned findings indicate that although the ultra-high plateau environment significantly influences human HRV during both day and night periods, it does not change the natural diurnal rhythm trend. Furthermore, the △HRV values were used to analyse and compare the diurnal variation amplitudes between the two groups. This study found that compared with the plain group, the ultra-high plateau group showed a significant increase in △LF and △LF/HF, and a significant decrease in △HF and △S. HF is widely used to characterize vagal nerve tension, and the results of this study show that the △HF value in the ultra-high plateau group was significantly lower than that in the plain group. This may suggest that the ultra-high plateau environment reduces the normal diurnal recovery ability of vagal nerve tension. In recent years, the significance of LF in indicating baroreflex sensitivity has garnered attention (Goldstein et al., 2011). Research by Sleight et al. (1995) indicates that carotid sinus stimulation induced by neck suction can increase LF in individuals with normal baroreflexes, whereas no increase in LF is observed in individuals with impaired baroreflexes. In this study, the significant increase in △LF in the ultra-high plateau group compared to the plain group may suggest that the ultra-high plateau environment excessively activates the body’s baroreceptors during the day-night transition, potentially leading to increased blood pressure. A clinical randomized trial has shown that 24-hour ambulatory blood pressure continuously rises with increasing altitude and decreases after lowering the altitude (Parati et al., 2014). Moreover, the increase in △LF and decrease in △HF observed in the ultra-high plateau group in this study led to an increased △LF/HF ratio. This can be explained from a mathematical logic perspective and also reflects changes in the balance of the ANS, namely, prolonged residence in the ultra-high plateau increases the inclination of ANS balance towards sympathetic nervous activity.

This study’s heart rate analysis indicates that both the mean and minimum heart rates in the ultra-high plateau group were significantly higher than those in the plain group. It has been reported that acute hypoxia can cause an increase in resting heart rate, which is associated with sympathetic nervous activation and a decline in parasympathetic nerve function (Koller et al., 1988). However, despite the gradual restoration of blood oxygen content with prolonged hypoxia and adaptation to the environment, the resting heart rate remains elevated (Naeije, 2010). Siebenmann et al. highlighted that the sustained elevation in heart rate during chronic hypoxia primarily stems from a persistent decrease in parasympathetic activity rather than continuous sympathetic stimulation (Siebenmann et al., 2017). This is consistent with our analysis of the overall 24-hour ANS function changes, showing a significant decline in vagal nerve tension accompanied by a significant increase in sympathetic nerve tension. Furthermore, the study observed a notably lower maximum heart rate in the ultra-high plateau group (see Table 5), echoing findings from ascents to extreme altitudes (Reeves et al., 1985). Chronic hypoxia leads to increased adrenaline spillover in healthy individuals (Calbet, 2003), resulting in continuous sympathetic nervous activation. Studies have shown that prolonged exposure to hypoxic conditions downregulates the density of cardiovascular adrenergic receptors (Kacimi et al., 1985a; Voelkel et al., 1981) and increases the expression of M2 muscarinic receptors (Kacimi et al., 1985b). This combined modulation of cardiac receptors could be one of the reasons for the decrease in maximum heart rate. Additionally, Richalet et al. have suggested that the ANS automatically regulates cardiovascular oxygen supply through this mechanism to prevent myocardial ischemia and potential hazardous events (Richalet and Hermand, 2022). Nonetheless, the increase in average and minimum heart rates caused by prolonged residence in the ultra-high plateau suggests a decline in cardiac reserve capacity and an increase in cardiac workload.

Recently, with in-depth research on HRV and its relationship with the ANS, scholars have linked HRV to sleep conditions, attempting to characterize sleep features through changes in HRV metrics (Cabiddu et al., 2012). In this study, we also attempted to analyze the sleep conditions of the healthy young population residing in the ultra-high plateau using HRV data. Findings indicate that, relative to the plain dwelling cohort, the ultra-high plateau residents exhibited increased awakenings, higher apnea-hypopnea index (AHI), and diminished sleep efficiency and quality. Such phenomena are prevalent in groups ascending to high-altitude locales (Weil, 2004), signifying potential sleep disturbances. In the high-altitude hypoxic environment, the gradual decrease in nighttime oxygen saturation and frequent occurrences of obstructive events caused by central sleep apneas lead to an increase in AHI and are somewhat related to the increased number of awakenings (Nussbaumer-Ochsner et al., 2010). Nevertheless, given that HRV analysis alone lacks the precision for definitive sleep condition assessment, these findings might only partly reflect the sleep dynamics of individuals in the ultra-high plateau.

Although HRV is an important indicator for assessing ANS function and is widely used to evaluate individual health status, it is influenced by multiple factors that cannot be ignored. Studies have shown that time-domain HRV measures, such as SDNN, SDANN, and the SDNN index, are not only higher in males but also gradually decline with age (Bonnemeier et al., 2003), whereas RMSSD and pNN50 exhibit a U-shaped pattern with increasing age (Almeida-Santos et al., 2016). A meta-analysis on sex differences in HRV indicated that females tend to exhibit a relative vagal dominance, while males show a relative sympathetic dominance (Koenig and Thayer, 2016). In this study, the age range of the included participants were within the young adult population. Therefore, the variations in HRV under ultra-high plateau conditions in other age groups remain to be further explored. Regarding sex differences, the study primarily included male participants, which may have influenced the final observations of sympathetic nervous activity indicators. Furthermore, whether male sympathetic nervous activity is more sensitive to altitude increases than that of females requires further investigation and validation in future research. In this study, there was an inter-group difference in BMI, but all participants were within a normal BMI range, with no instances of overweight or obesity. Although no correlation between HRV and BMI was found in a study with a sample of 653 individuals without heart disease (Antelmi et al., 2004), the relationship between BMI and HRV in ultra-high plateau environments still requires further investigation. Autonomic dysfunction is linked to various pathophysiological conditions, including hypertension (Lucini et al., 2014; Huikuri et al., 1996), diabetes mellitus (Gerritsen et al., 2001), and others. The Framingham Heart Study demonstrated that lower HRV is associated with an increased risk of hypertension in men with normal blood pressure (Singh et al., 1998). Similarly, the Atherosclerosis Risk in Communities (ARIC) study revealed that during a 9-year follow-up, participants in the lowest RMSSD quartile had a 1.36-fold higher risk of developing hypertension compared to those in the highest quartile (Schroeder et al., 2003). A study by Mori et al. indicated that a decrease in HRV is associated with an increase in blood pressure, significantly affecting diastolic blood pressure levels in both genders (Mori et al., 2014). A meta-analysis highlighted that type 2 diabetes mellitus is associated with an overall reduction in HRV, with both sympathetic and parasympathetic nervous tone diminished in patients with type 2 diabetes mellitus (Benichou et al., 2018). In our study, participants from the ultra-high plateau group exhibited lower overall HRV, elevated sympathetic nerve tone, and reduced parasympathetic nerve tone compared to those from plain areas. This suggests that individuals from plain areas living in ultra-high plateau environments for prolonged periods may have an increased risk of developing hypertension and diabetes. The relationship between ultra-high plateau environments and the body’s pathophysiological state, as well as the associated increased disease risk, warrants further research.

In summary, prolonged residence in the ultra-high plateau environment will significantly decrease HRV in the healthy young population, with ANS showing reduced vagal activity accompanied by sustained increase in sympathetic activity; more importantly, although prolonged residence in the ultra-high plateau does not change the intrinsic circadian rhythm trend of the young healthy population, it reduces their vagal recovery ability during the circadian cycle and causes an imbalance in the ANS towards increased sympathetic activity. Additionally, the ultra-high plateau environment not only reduces the cardiac reserve function of the healthy young population but also decreases their sleep quality. HRV, as a convenient and non-invasive index, can provide accurate guidance on the health status of people residing in high-altitude areas, thus effectively preventing the occurrence of high-altitude risks. Of course, the variations in HRV in various diseases, especially the characteristics in high-altitude illnesses, still need to be further explored.

## 5 Conclusion

In summary, our study found that prolonged residence in the ultra-high plateau reduces HRV, cardiac reserve capacity, and sleep quality in healthy young adults, diminishes the diurnal recovery capacity of the vagal nerve, and leads to a shift in ANS balance towards reduced vagal activity and enhanced sympathetic activity.

## 6 Limitations

The limitations of this study are as follows: 1) The subjects were primarily male, which may lead to a gender bias in the data; 2) Polysomnography (PSG) is the gold standard for assessing sleep phases, and the subjects did not undergo PSG measurements; 3) The subject group did not undergo a comprehensive assessment of physiological indicators. In future experiments, we will address these shortcomings by including gender, PSG, and physiological parameters in our research plan to fully assess the role of HRV.

## Data Availability

The original contributions presented in the study are included in the article/supplementary material, further inquiries can be directed to the corresponding authors.
